# Supplementary material for: Characterization and Biological Activities of Four Biotransformation Products of Diosgenin from Rhodococcus erythropolis
Source: Molecules. 2023 Mar 30;28(7):3093. doi: 10.3390/molecules28073093 (PMC10096415; doi:10.3390/molecules28073093)
Supplement: Supplementary file 1 [file molecules-28-03093-s001.zip › molecules-2191262-supplementary.pdf]

molecules

Supporting Information

Article

Characterization and Biological Activities of Four Biotransformation Products of Diosgenin from *Rhodococcus erythropolis*

Yanjie Li [1,2], Chengyu Zhang [1,2], Kexin Kong [1] and Xiaohui Yan [1,2]*

[1] State Key Laboratory of Component-based Chinese Medicine, Tianjin University of Traditional Chinese Medicine, 10 Poyanghu Road, Jinghai District, Tianjin, 301617, China
[2] Haihe Laboratory of Modern Chinese Medicine, 10 Poyanghu Road, Jinghai District, Tianjin, 301617, China
* Correspondence: yanxh@tjutcm.edu.cn.

Table S1. ^{1}H (600 MHz) and ^{13}C NMR (150 MHz) spectroscopic data of compounds **1** and **2** in $CDCl_3$

	**1**		**2**	
Position	δ_C[a]type	δ_H[b](J in Hz)	δ_C[a]type	δ_H[b](J in Hz)
1	35.7	2.01 (1H, m); 1.69 (1H, m)	155.7	7.04 (1H, d, J = 10.1)
2	34	2.35 (1H, m); 2.41(1H, m)	127.6	6.23 (1H, dd, J = 10.1, 1.8)
3	199.5	-	186.3	-
4	123.9	5.72 (1H, s)	123.9	6.07 (1H, s)
5	171.2	-	169	-
6	32.8	2.27 (1H, m); 2.41 (1H, m)	32.8	2.36(1H, m); 2.47 (1H, m)
7	32.1	1.04 (1H, m); 1.87 (1H, m)	31.9	1.34 (1H, m); 2.00 (1H, m)
8	35.2	1.72 (1H, m)	35.2	1.82 (1H, m)
9	53.8	0.95 (1H, m)	52.4	1.08 (1H, m)
10	38.7	-	43.6	-
11	20.8	1.44 (1H, m); 1.53 (1H, m)	22.7	1.67 (1H, m); 2.00 (1H,m)
12	39.7	1.17 (1H, m); 1.75 (1H, m)	39.5	1.18 (1H, m); 1.78 (1H, m)
13	40.4	-	40.7	-
14	55.7	1.12 (1H, m)	55.3	1.12 (1H, m)
15	31.7	1.31 (1H, m); 2.01 (1H, m)	33.8	1.07 (1H, m); 1.96 (1H, m)
16	80.6	4.40 (1H, dd, J = 15.2, 7.7)	80.5	4.40 (1H, dd, J = 15.1, 7.7)
17	62	1.77 (1H, m)	62	1.77 (1H, m)
18	16.4	0.82 (3H, s)	16.4	0.85 (3H, s)
19	17.4	1.19 (3H, s)	18.8	1.25 (3H, s)
20	41.7	1.87 (1H, t, J = 6.9)	41.7	1.87 (1H, dd, J = 6.9)
21	14.5	0.97 (3H, d, J = 6.9)	14.5	0.97 (3H, d, J = 7.0)
22	109.3	-	109.3	-
23	30.3	1.69 (2H, m)	30.3	1.69 (2H, m)
24	28.8	1.60 (2H, m)	28.8	1.59 (2H, m)
25	31.4	1.71 (1H, m)	31.4	1.71 (1H, m)
26	66.9	3.46 (1H, m); 3.36 (1H, m)	66.9	3.48 (1H, m); 3.36 (1H, m)
27	17.1	0.79 (3H, d, J = 6.4)	17.1	0.79 (3H, d, J = 6.4)

δ in ppm, J in Hz. s-singlet; d-doublet; t-triplet; m-multiple

Table S2. Crystal data and structure refinement

Crystallographic data	Compound 4
Empirical formula	$C_{27}H_{36}O_5$
Formula weight	440.56
Temperature/K	170.00
Crystal system	orthorhombic
Space group	$P2_12_12$
a/Å	16.3270(9)
b/Å	20.3577(11)
c/Å	7.0798(4)
α/°	90
β/°	90
γ/°	90
Volume/Å^3	2353.2(2)
Z	4
ρ_{calc}g/cm^3	1.244
μ/mm^{-1}	0.430
F(000)	952.0
Crystal size/mm^3	0.2 × 0.12 × 0.08
Radiation	GaKα (λ = 1.34139)
2Θ range for data collection/°	6.036 to 121.19
Index ranges	-21 ≤ h ≤ 21, -26 ≤ k ≤ 26, -8 ≤ l ≤ 9
Reflections collected	38910
Independent reflections	5390 [R_{int} = 0.0416, R_{sigma} = 0.0240]
Data/restraints/parameters	5390/0/293
Goodness-of-fit on F^2	1.052
Final R indexes [I>=2σ (I)]	R_1 = 0.0331, wR_2 = 0.0887
Final R indexes [all data]	R_1 = 0.0344, wR_2 = 0.0898
Largest diff. peak/hole / e Å^{-3}	0.39/-0.34
Flack parameter	-0.04(5)
CCDC Deposition N.	2245739

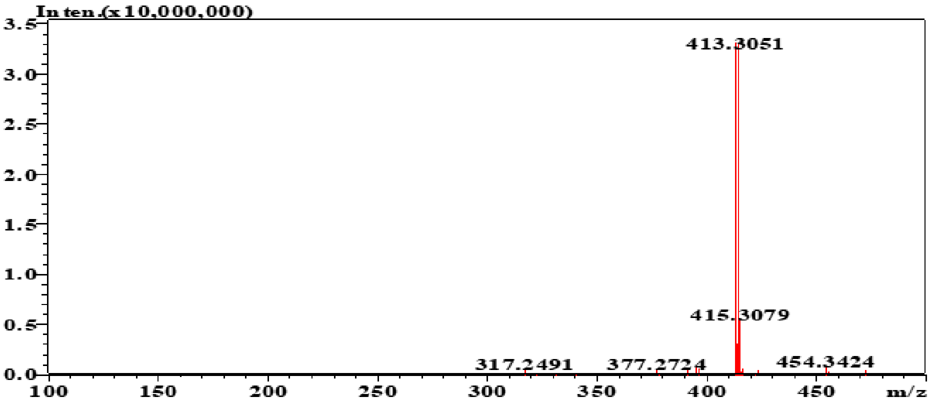

Figure S1. MS spectrum of compound **1**

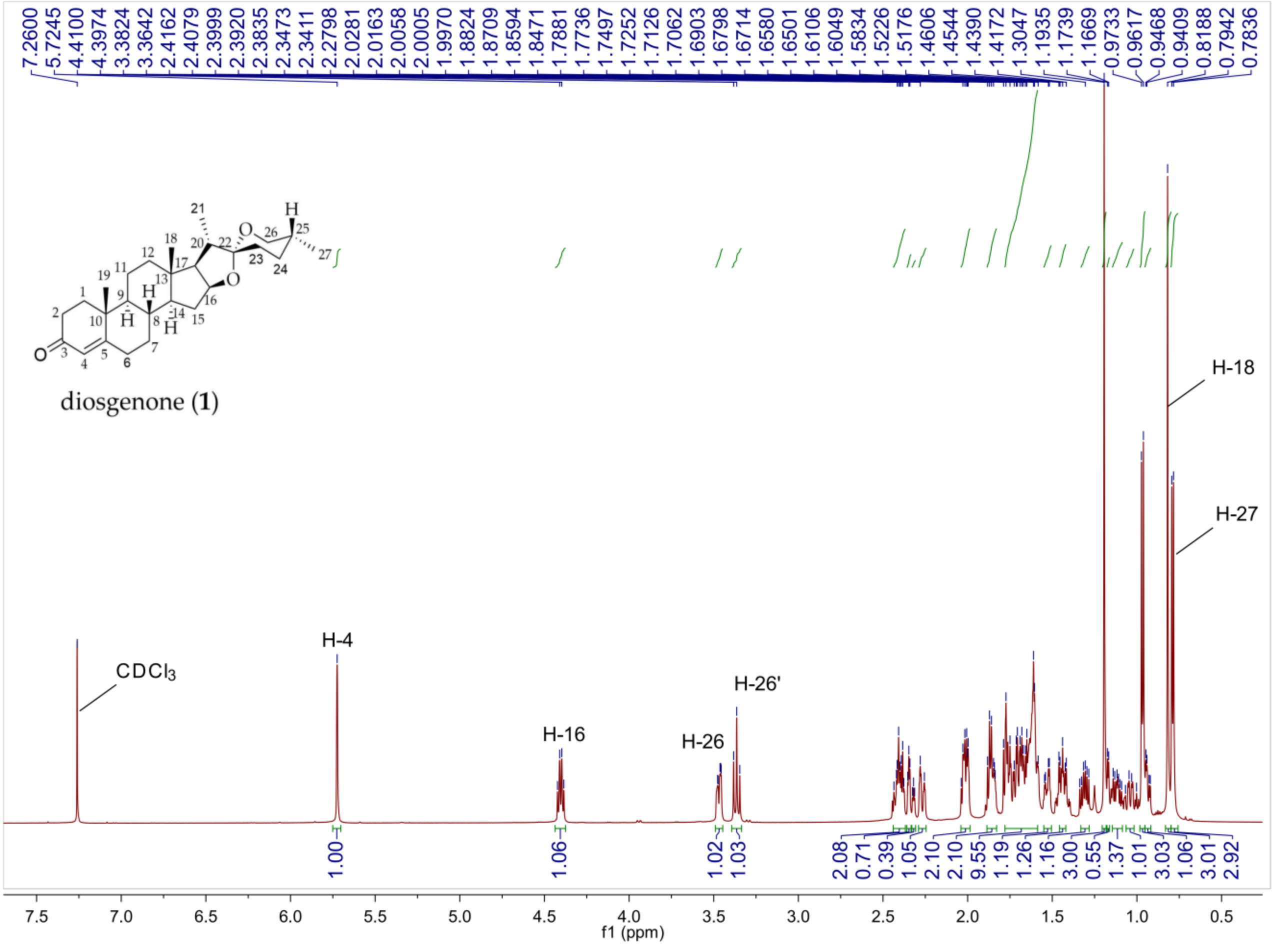

Figure S2. ^{1}H-NMR spectrum of compound **1** (600 MHz, in $CDCl_3$)

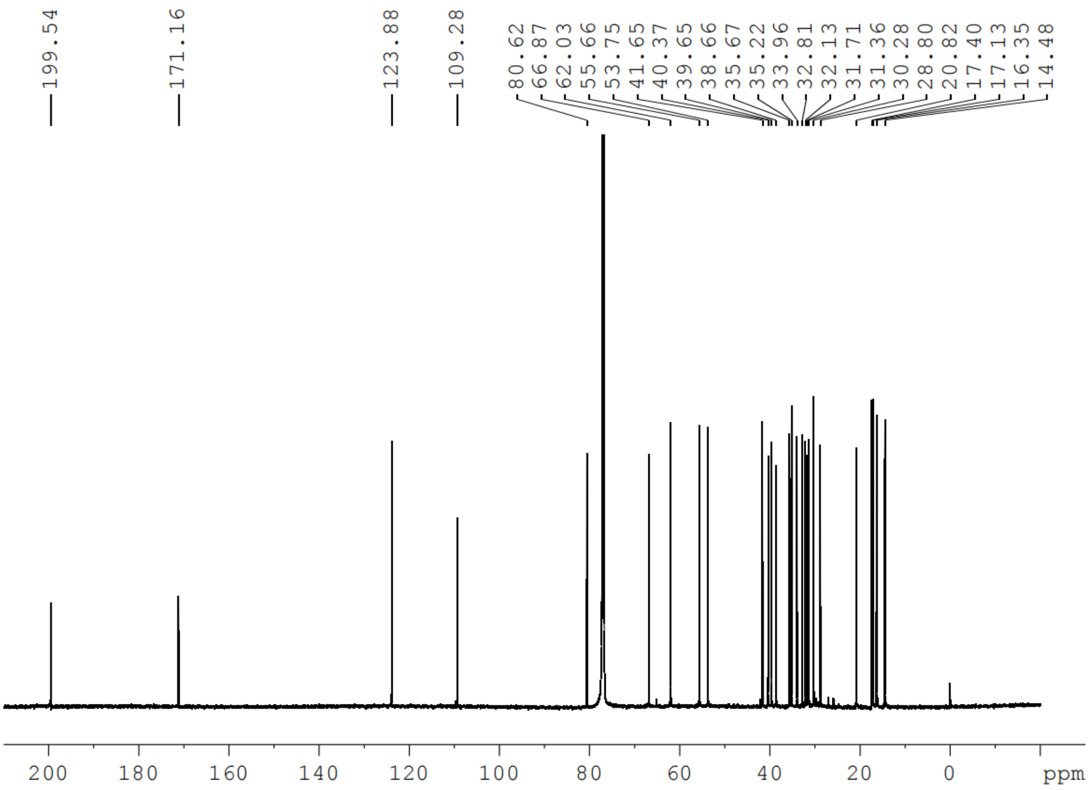

Figure S3. ^{13}C-NMR spectrum of compound **1** (150 MHz, in $CDCl_3$)

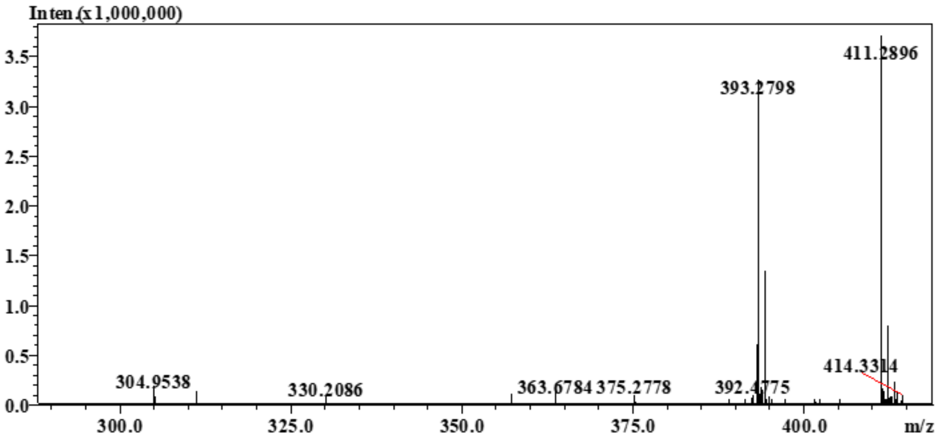

Figure S4. MS spectrum of compound **2**

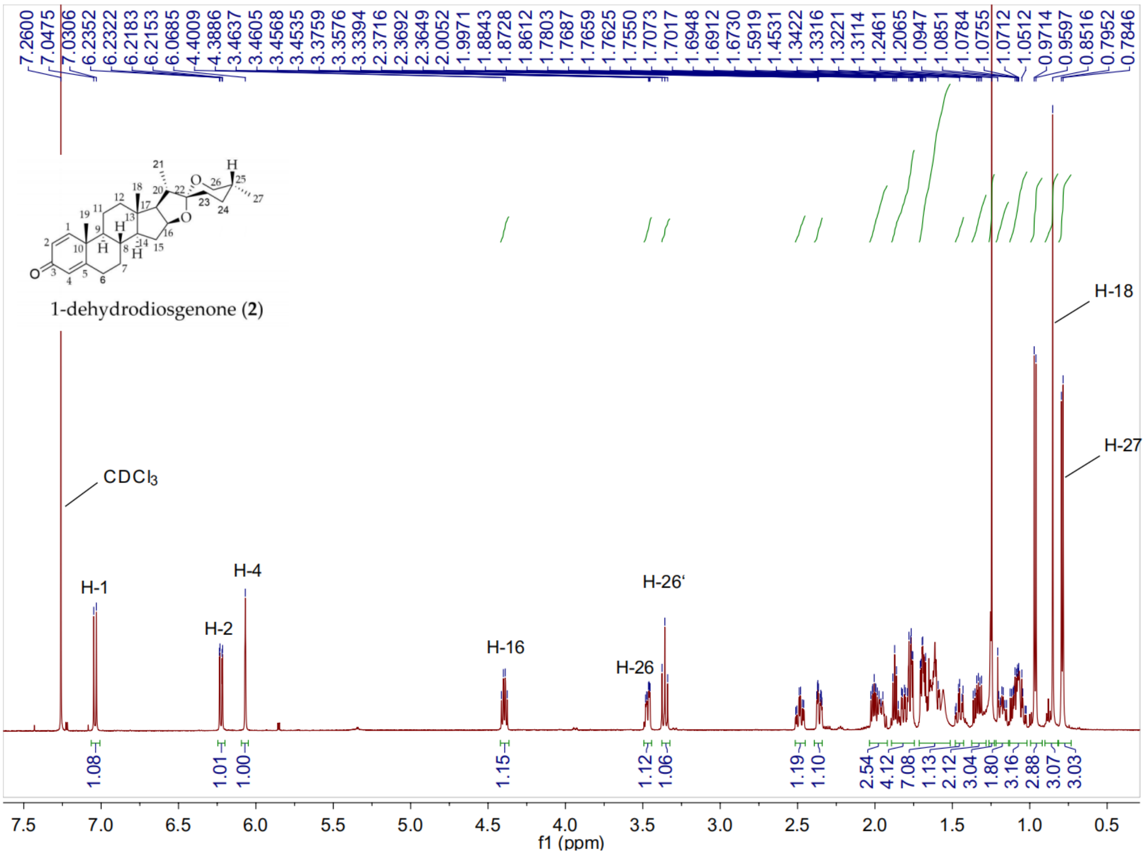

Figure S5. ^{1}H-NMR spectrum of compound **2** (600 MHz, in $CDCl_3$)

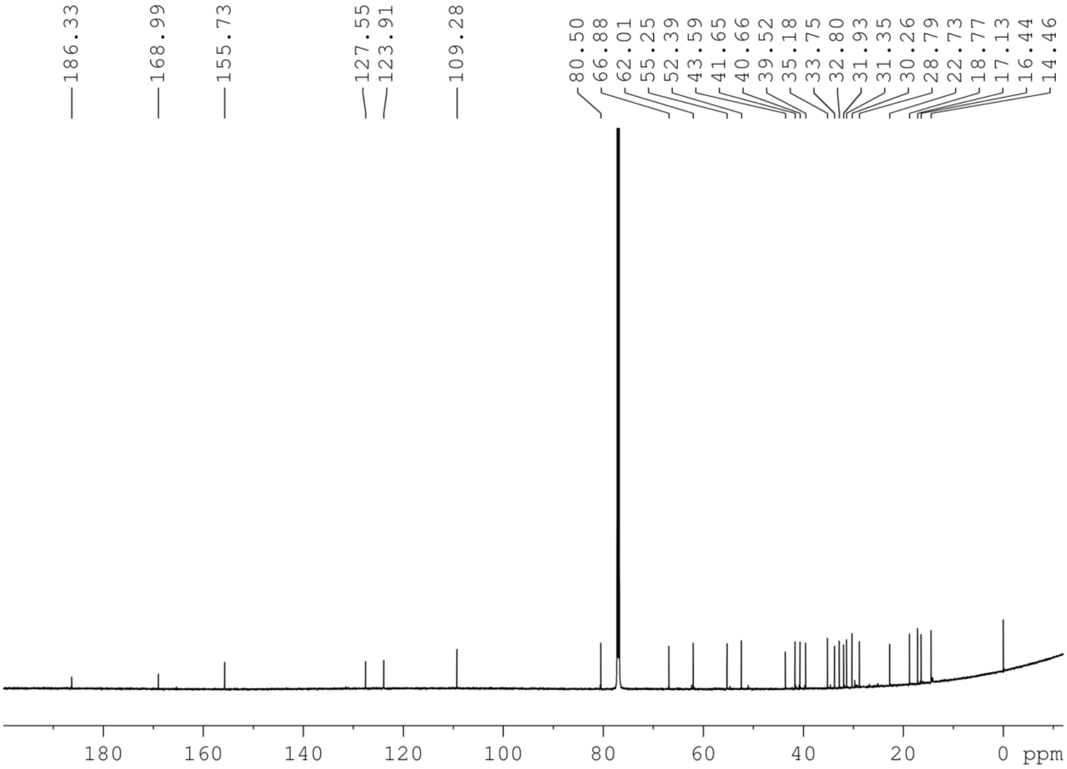

Figure S6. ^{13}C-NMR spectrum of compound **2** (150 MHz, in $CDCl_3$)

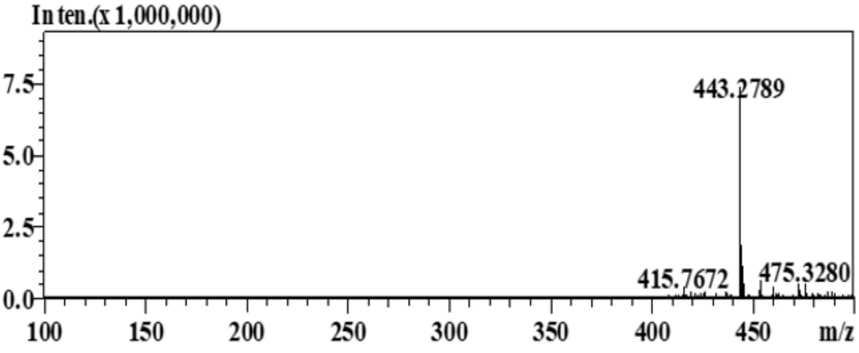

Figure S7. MS spectrum of compound **3**

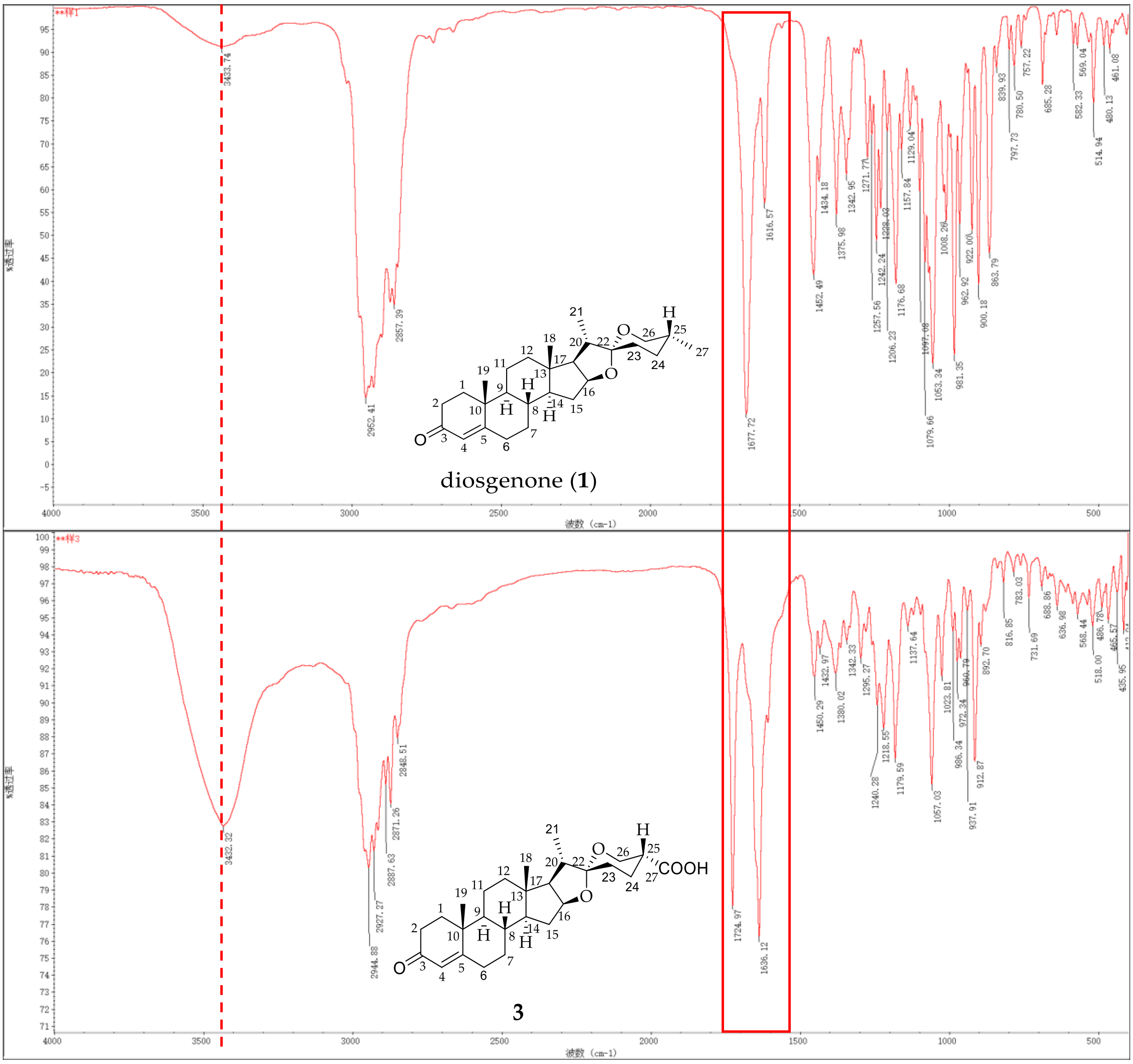

Figure S8. IR spectrum of compounds **1** and **3**

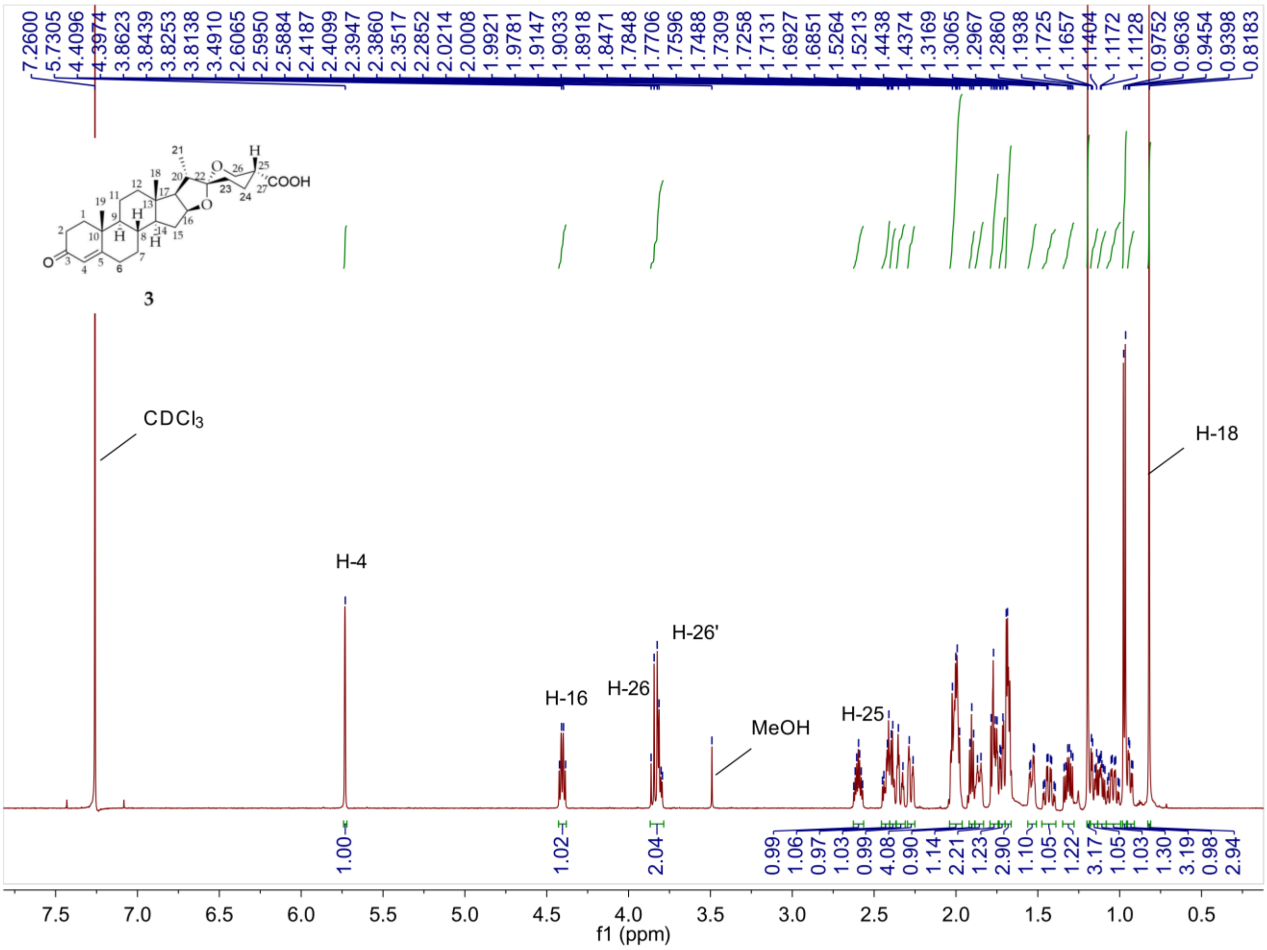

Figure S9. ^{1}H-NMR spectrum of compound **3** (600 MHz, $CDCl_3$)

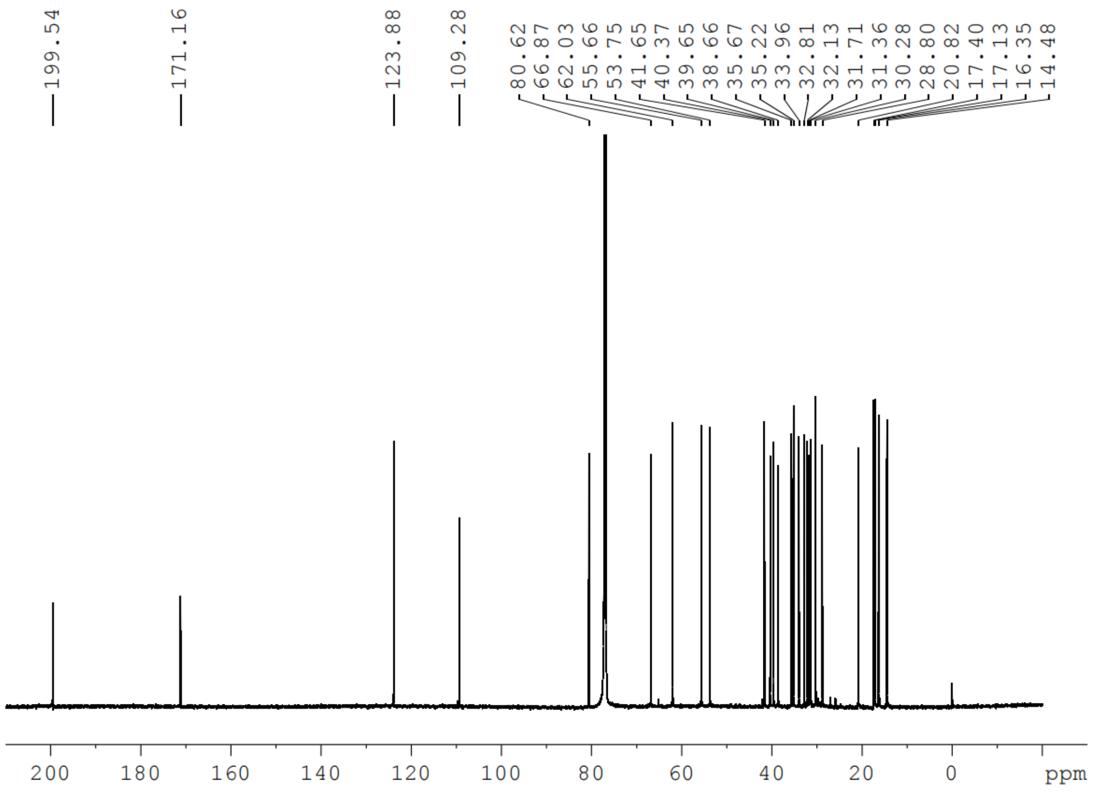

Figure S10. ^{13}C-NMR spectrum of compound **3** (150 MHz, $CDCl_3$)

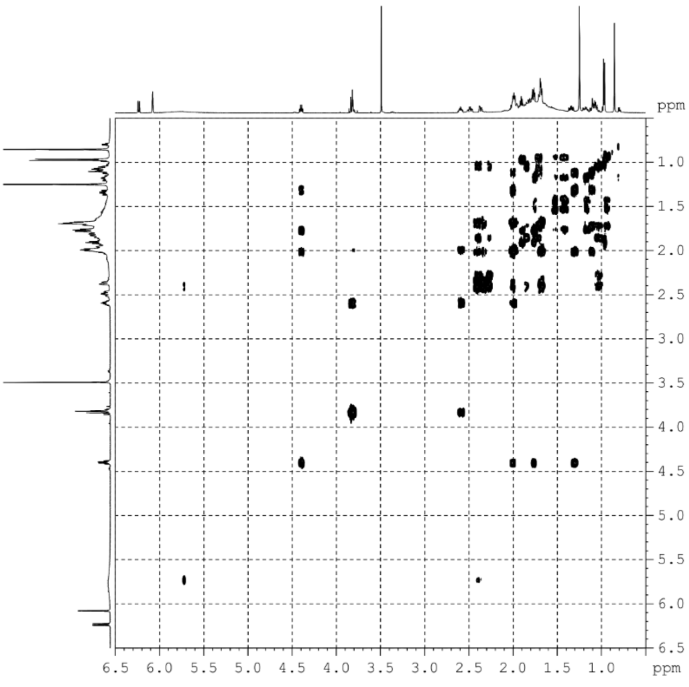

Figure S11. ^{1}H–^{1}H COSY spectrum of compound **3** ($CDCl_3$)

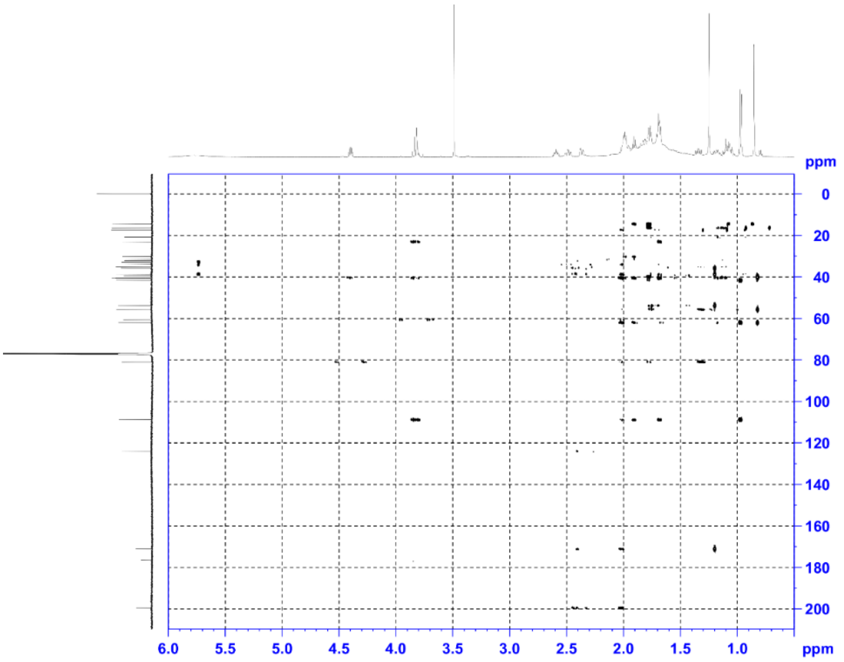

Figure S12. HMBC spectrum of compound **3** ($CDCl_3$)

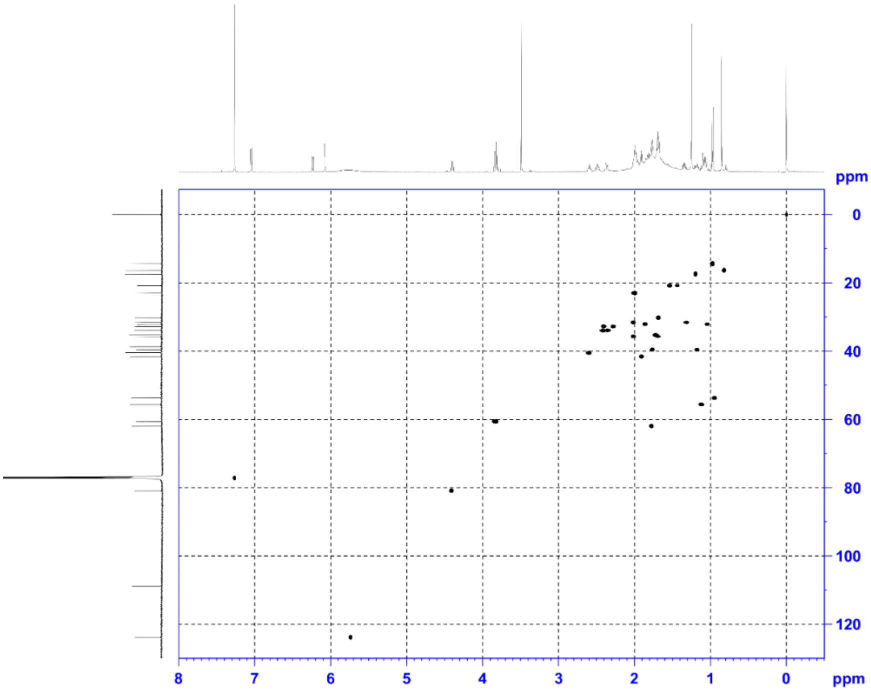

Figure S13. HSQC spectrum of compound **3** ($CDCl_3$)

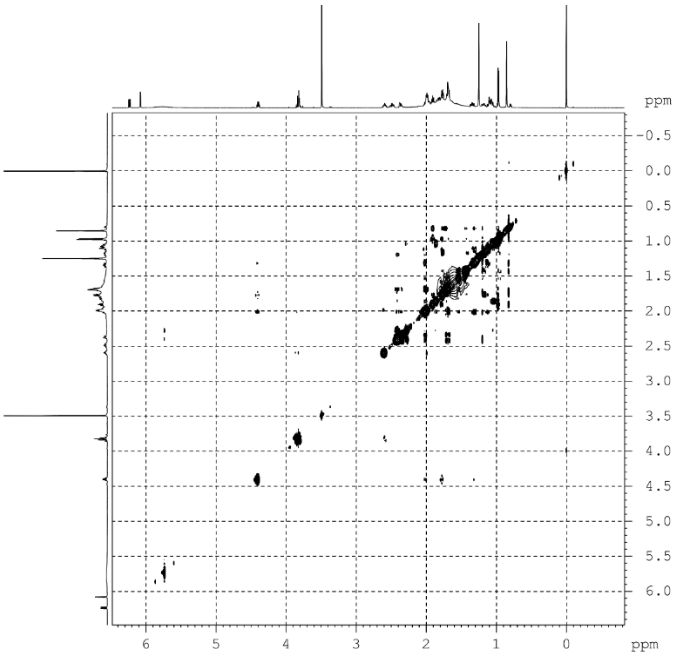

Figure S14. NOESY spectrum of compound **3** ($CDCl_3$)

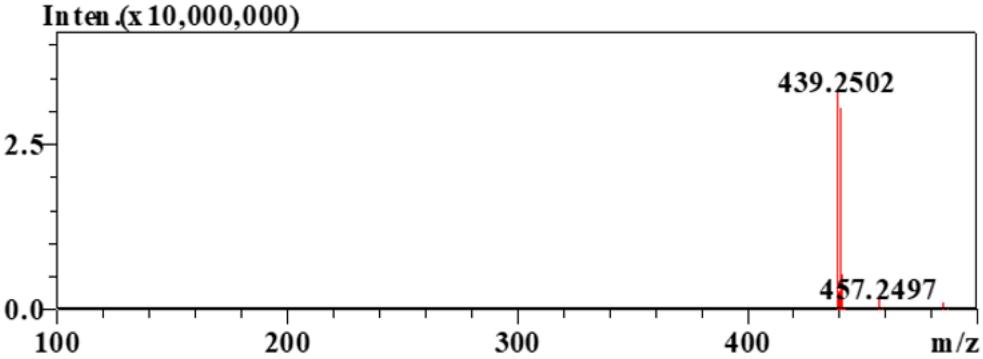

Figure S15. MS spectrum of compound **4**

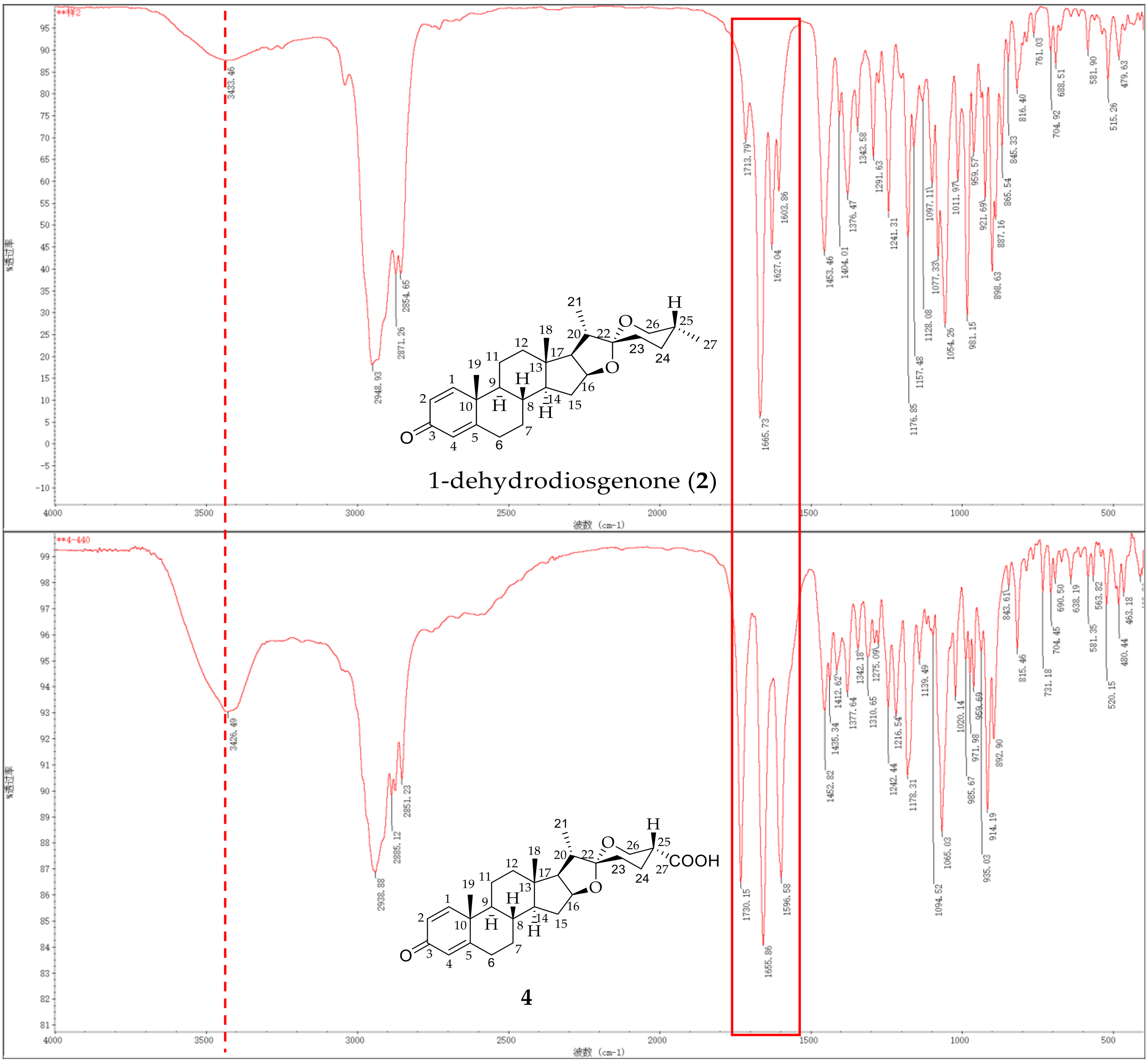

Figure S16. IR spectrum of compound **2** and **4**

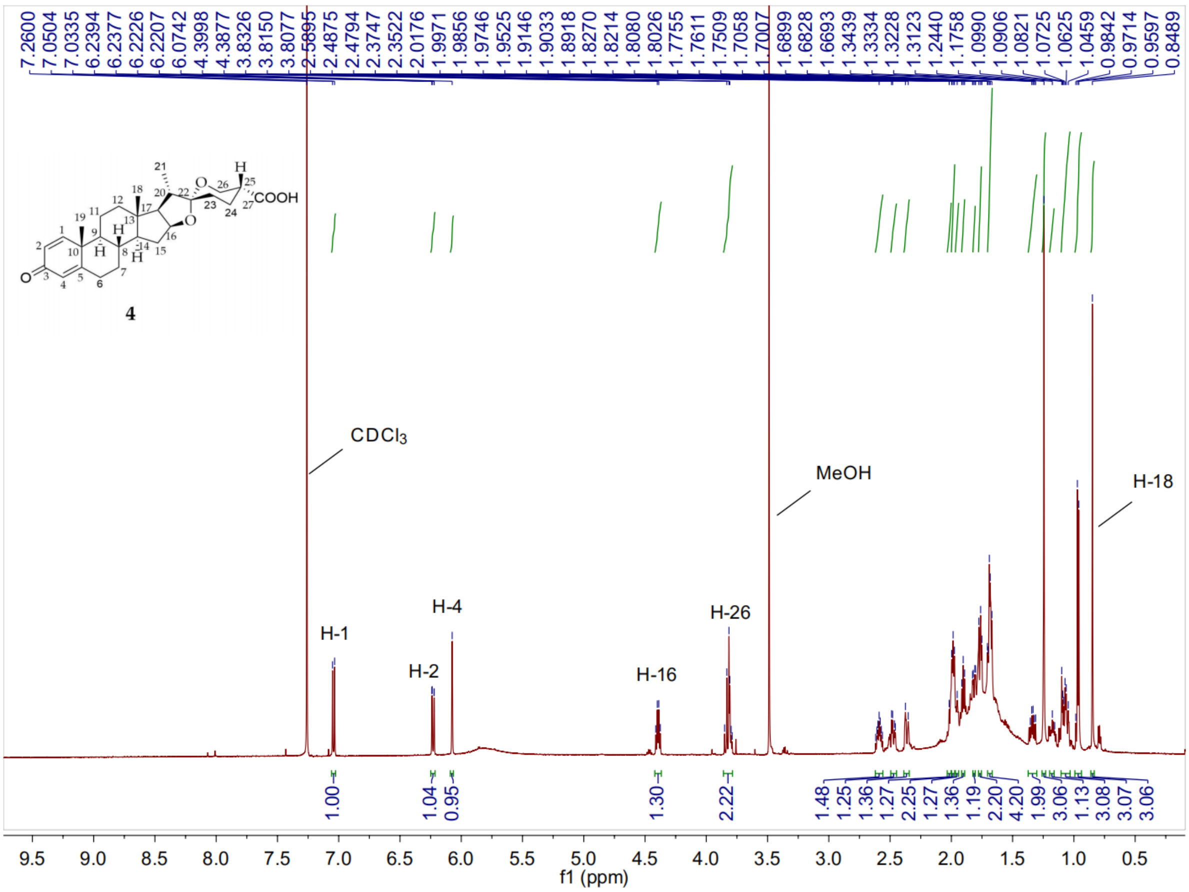

Figure S17. ^{1}H-NMR spectrum of compound **4** (600 MHz, $CDCl_3$)

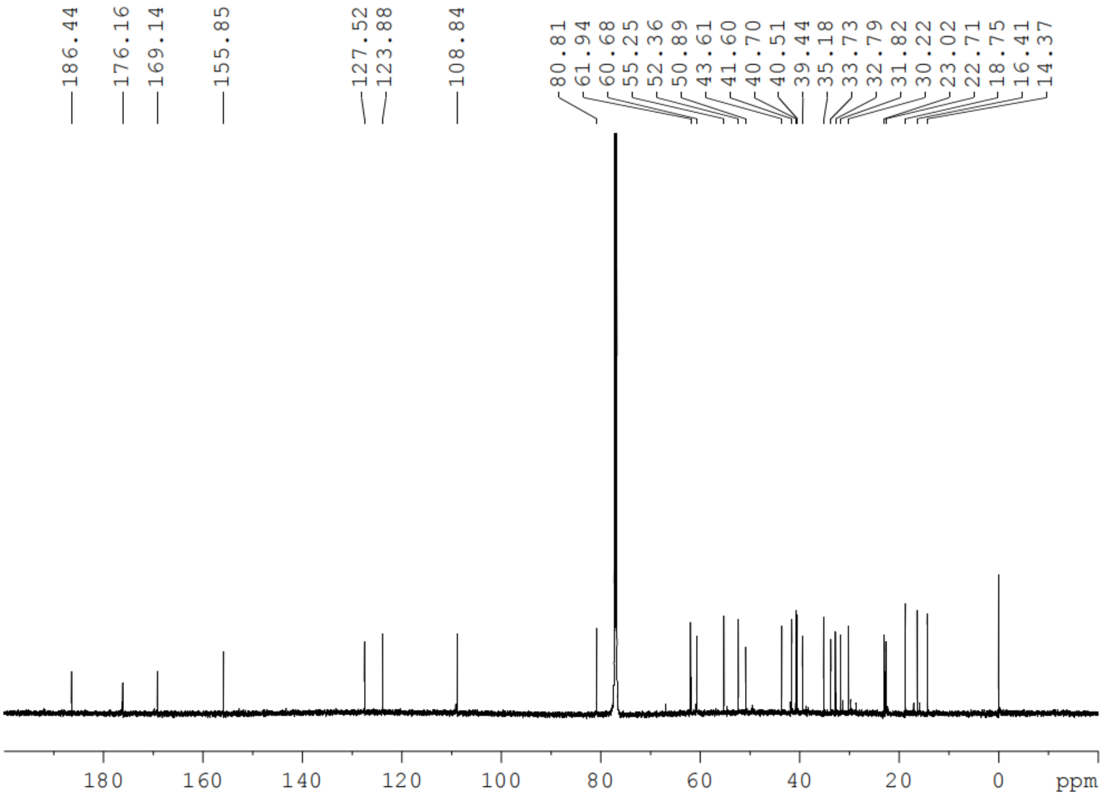

Figure S18. ^{13}C-NMR spectrum of compound **4** (150 MHz, $CDCl_3$)

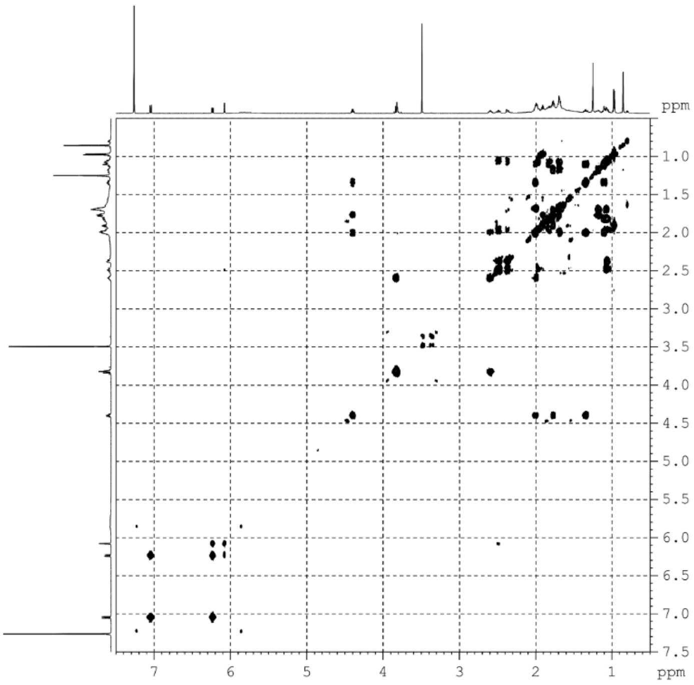

Figure S19. ^{1}H–^{1}H COSY spectrum of compound **4** ($CDCl_3$)

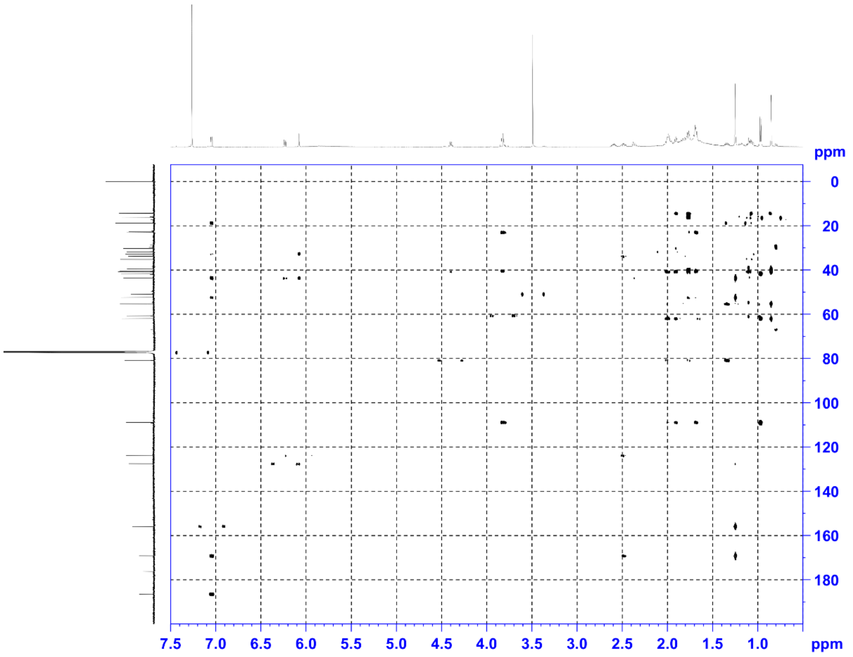

Figure S20. HMBC spectrum of compound **4** ($CDCl_3$)

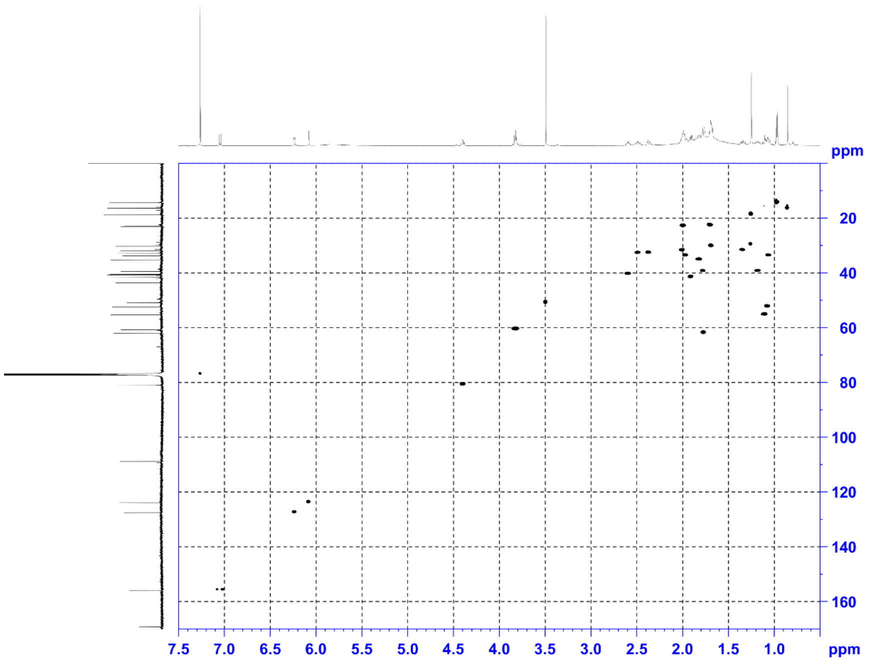

Figure S21. HSQC spectrum of compound **4** ($CDCl_3$)

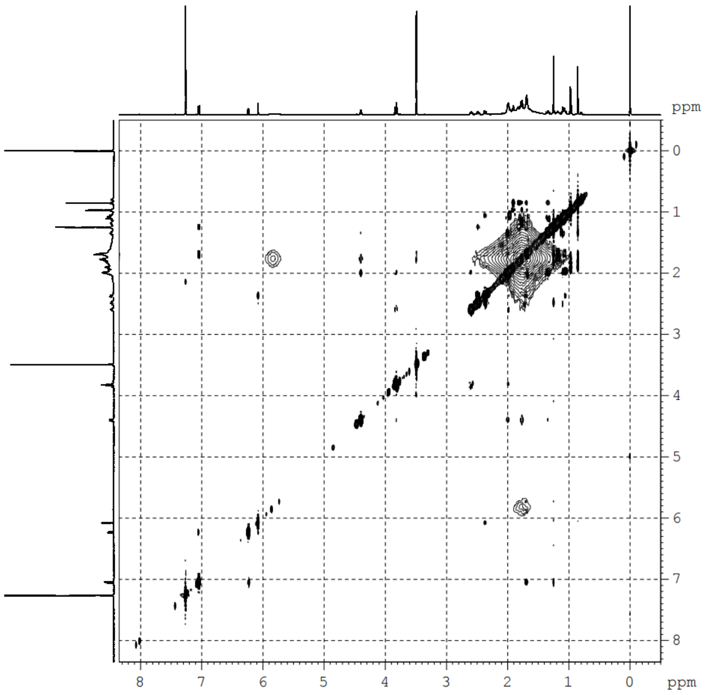

Figure S22. NOESY spectrum of compound **4** ($CDCl_3$)

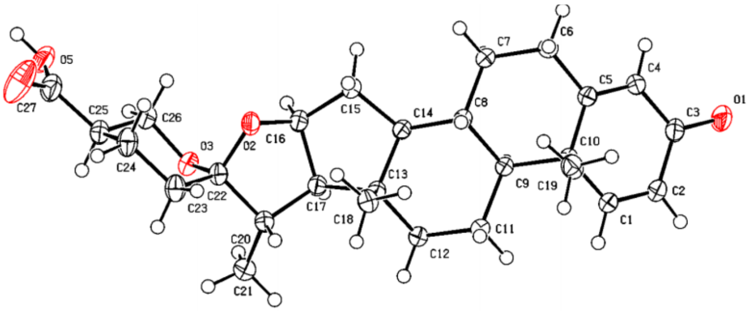

Figure S23. X-ray crystal structure of compound **4**

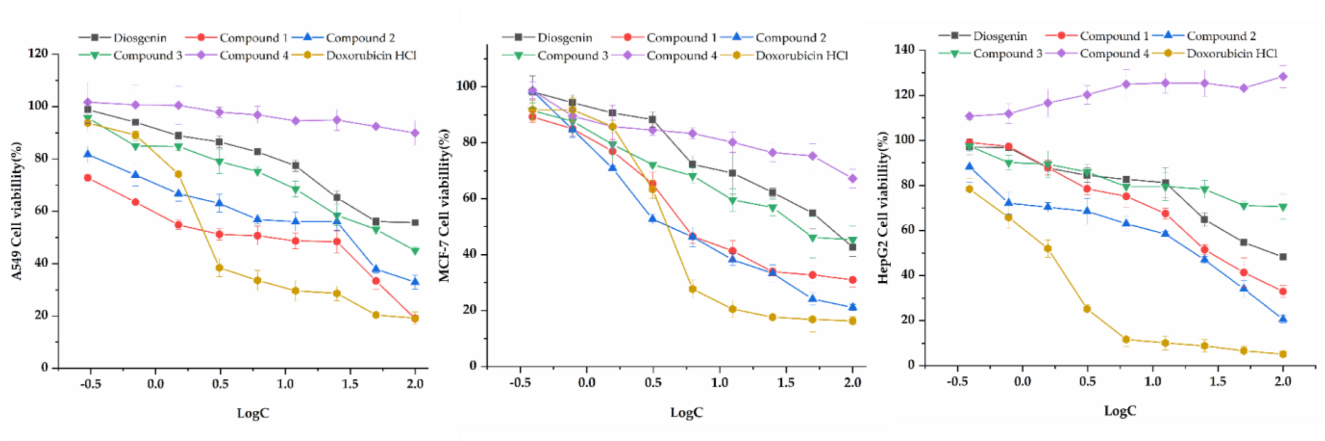

Figure S24. Effect of DSG and compounds **1–4** on the survival rate of A549, MCF-7, and HepG2 cells

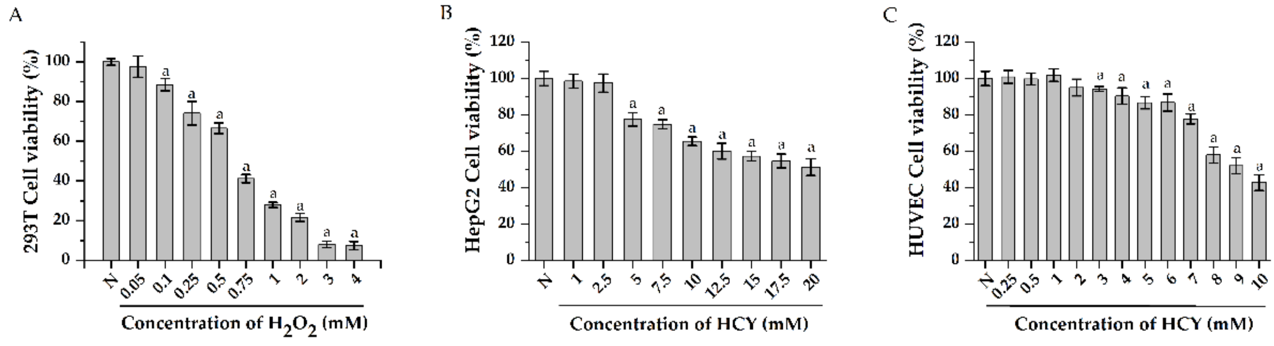

Figure S25. Effect of different concentrations of H_2O_2 or Hcy on the cell viability of (A) 293T, (B) HepG2, and (C) HUVEC cells. Note: [a]P are compared with the normal group, extremely significant difference ($p < 0.01$).

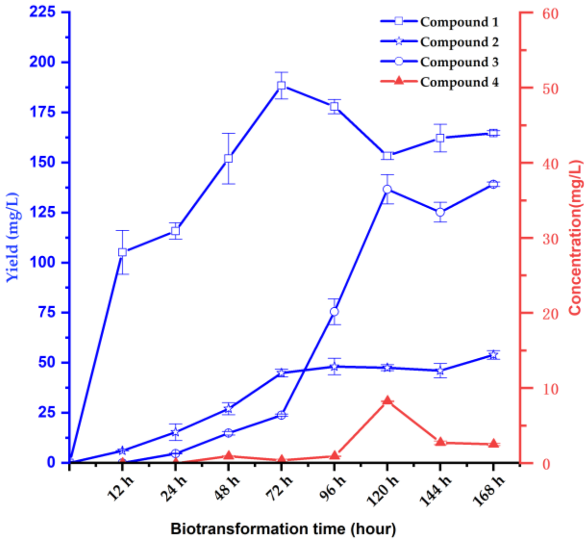

Figure S26. Time course of biotransformation. Changes in yields of biotransformation products **1–4** from 12 h to 7 days of incubation

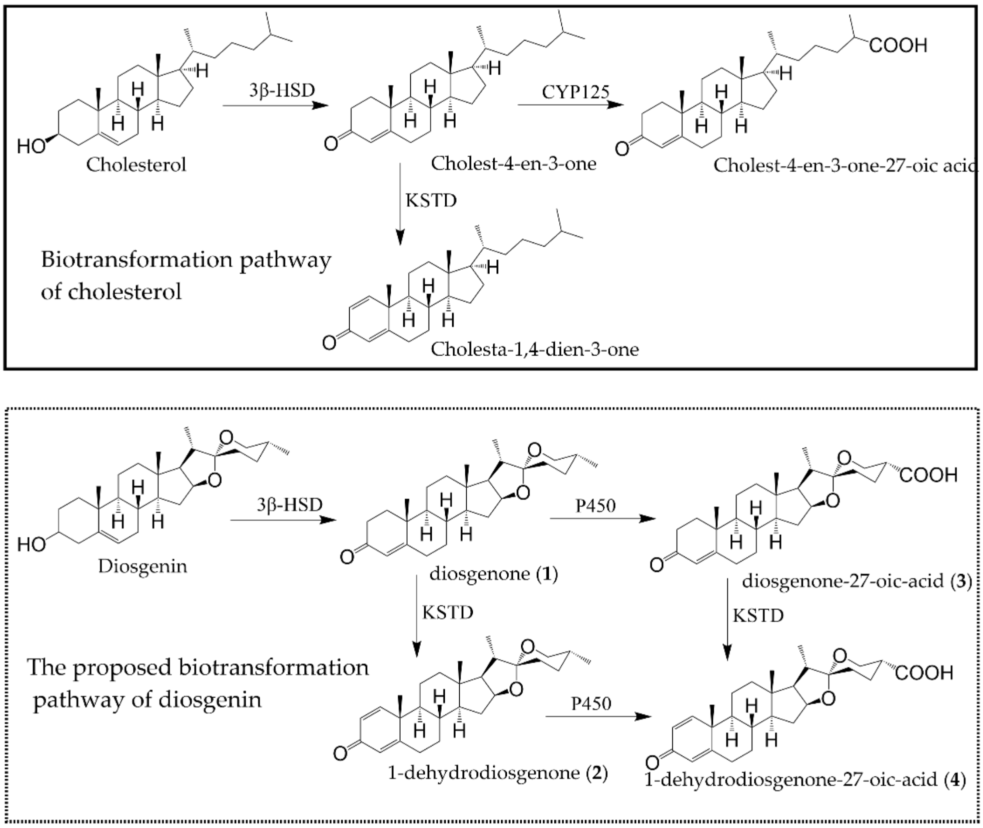

Figure S27. Proposed the metabolic pathway of DSG in *R. erythropolis* based on the metabolic pathway of cholesterol
